# Assessing the robustness of normality tests under varying skewness and kurtosis: a practical checklist for public health researchers

**DOI:** 10.1186/s12874-025-02641-y

**Published:** 2025-09-01

**Authors:** Asha Kamath, Satyanarayana Poojari, K. Varsha

**Affiliations:** Department of Applied Statistics and Data Science, Prasanna School of Public Health, Manipal Academy of Higher Education Manipal, Udupi, Karnataka India

**Keywords:** Normality, Sample size, Type 1 error rate, Power, Skewness and Kurtosis

## Abstract

**Background:**

Many statistical methods used in public health research, namely t-tests, ANOVA correlation and regression, rely on the assumption of normality. Violation of the normality assumption can severely lead to biased parameter estimates, reduced test power, and impact the reliability and validity of the findings, impacting the real-world evidence. An attempt to provide guidelines for choice of appropriate tests for assessment in public health data analytics is being made in this article.

**Methods:**

This study aims to compare the performance of 13 commonly available normality tests in various software’s, namely Shapiro–Wilk, Shapiro-Francia (Regression-Based tests), Lilliefors, Cramer Von Mises, Anderson–Darling (Empirical distribution-based test), Jarque–Bera, Adjusted Jarque Bera Test, Robust Jarque–Bera, D’Agostino & Pearson, D’Agostino Skewness, D’Agostino Kurtosis, Gel Miao Gastwirth (Moment-Based test), and Pearson Chi-Square (Chi-square-based test). These tests were evaluated based on empirical Type I error and power across varying sample sizes, skewness, and kurtosis using Monte Carlo simulations with non-normal data generated via the Fleishman method, reflecting slight to significant deviations in terms of skewness and kurtosis.

**Results:**

For moderately skewed data with low kurtosis, the D’Agostino Skewness and Shapiro–Wilk tests perform better across all sample sizes while Robust and Adjusted Jarque–Bera tests are preferable at higher kurtosis. In highly skewed data, Shapiro–Wilk is most effective, with Shapiro-Francia and Anderson–Darling improving with larger samples. For symmetric data, RJB and GMG are robust choices, with GMG preferred at higher kurtosis. Findings from two real-world datasets also support the simulation results.

**Conclusion:**

Performance of Normality tests are significantly influenced by sample size, skewness, and kurtosis. The findings of this study contribute to improving statistical practices in public health research by providing a practical, evidence-based checklist for selecting appropriate normality tests based on these key sample characteristics.

## Background

When dealing with continuous data in public health research, assessing normality is a critical preliminary step in selecting appropriate measures of central tendency and statistical analysis techniques. In public health research, where t-tests, ANOVA, correlation and regression analysis methods are frequently used to analyze clinical and epidemiological data. These methods are built on the assumption of normally distributed data or residuals, which ensures validity, reliability, and generalizability of study findings (Lumley et al.,2001) [21]. However, if the assumption is not met, nonparametric methods may be more appropriate. Evaluating the distribution of data enhances the precision of estimates and the credibility of inferences, which is particularly important in studies that inform public health interventions, policy development, and the allocation of healthcare resources. Therefore, checking for normality is not only a technical requirement but also a foundational component of maintaining methodological accuracy and ensuring the production of evidence-based public health decisions.

Several researchers have highlighted the challenges posed by non-normality in statistical inference. Huber (1973) [13] demonstrated that, under non-normal conditions, it becomes difficult to establish necessary and sufficient conditions for the asymptotic normality of all parameter estimates. Koenker (1982) [19] further emphasized that the performance of commonly used t and F tests is highly sensitive to the assumed distribution; their power can deteriorate rapidly in the presence of long-tailed distributions. Bera and Jarque (1982) [14] also found that standard tests for homoscedasticity and serial independence, which are appropriate under normality, may lead to incorrect conclusions when applied to non-normal data. In real-world settings, sample data often follow non-normal distributions more frequently than normal distributions [6]. When the normality assumption is violated, it can lead to biased parameter estimates (Knief and Forstmeie, 2021 [18], Shatz, 2024 [31]) and misleading confidence intervals or *p*-values. These issues are particularly problematic in studies with small sample sizes. Furthermore, skewed or heavy-tailed distributions common in health-related data such as healthcare costs, hospital stays, or biomarker levels require careful handling to avoid erroneous conclusions. If non-normality is not properly detected, the results of statistical analyses may be misleading, compromising the credibility of the findings and potentially leading to flawed policy decisions or ineffective public health interventions. Therefore, assessing the robustness of normality tests under non-normal conditions is essential to ensure the integrity of statistical conclusions in public health research.

There are three common methods for checking normality: graphical, numerical, and formal tests. Graphical methods involve techniques like normal quantile–quantile (Q-Q) plot, histogram, box plot, and stem-and-leaf plot. Numerical methods include calculating skewness and kurtosis coefficients, while formal tests involve statistically evaluating whether the data follows a normal distribution. Even though graphical methods are useful for visually assessing normality, they are subjective. Proper interpretation requires vast experience and strong statistical knowledge, making these methods insufficient on their own for conclusively determining normality. Therefore, it is recommended to supplement graphical methods with descriptive statistics and formal normality tests for a more reliable assessment [24].

Numerous tests in literature evaluate normality, each with its strengths and application contexts. Many of these tests are commonly available in popular statistical software and are widely applied in real-life practice. Starting with Pearson [26] and the introduction of the chi-square test, many normality tests have been introduced and later modified to optimize their performance. Cramer [3] and Mises [22] introduced the Cramer-Von Mises tests which is a goodness of fit test based on squared differences between the empirical Cumulative Distribution Function (CDF) of the sample and CDF of the hypothesized distribution. Stephens [33] later modified this test by giving more weightage to the tails of the distribution and proposed the Anderson–Darling test. The critical values of the famous Kolmogorov–Smirnov test were modified by Lilliefors [20] to accommodate situations when distribution is not completely specified thus proposing the Lilliefors test. Additionally, Shapiro et al. [29] introduced the Shapiro–Wilk test, whose test statistic computes the correlation between the observed data and the corresponding normal scores. The test statistic involves computation of the covariance matrix which is extremely time consuming for large datasets. To solve this issue Shapiro and Francia [30] introduced the Shapiro Francia test. D’Agostino [4] introduced the D’Agostino-skewness test based on the normalization transformation of the skewness test (Shapiro et al. [29]) and further combining the transformations for skewness and kurtosis D’Agostino and Pearson [5] introduced the omnibus D’Agostino Pearson test. Later, Jarque and Bera [15] proposed the Jarque–Bera test based on sample skewness and kurtosis. Urzua [36] improvised the Jarque Bera test to improve its chi-square approximation in finite samples and proposed the Adjusted Jarque Bera test. A test for detecting normality, particularly in heavy-tailed observations was developed by Gel, Miao, and Gastwirth [12] called the Gel-Miao-Gastwirth test. Further in 2008, by using the robust estimate of dispersion in skewness and kurtosis they improvised the Jarque Bera test and proposed the Robust Jarque Bera test.

Apart from the above-mentioned commonly used tests for normality, several other tests are available and continue to be developed in literature. Evaluating the performance of these tests under different conditions provides a clearer understanding of their reliability and applicability in practical cases. For instance, in regression analysis, residual normality can accommodate slight deviations from normality when the sample size is sufficiently large while still ensuring reliable statistical inferences. However, the normality tests could result in significant results even for small deviations from normality for large sample sizes (Field and Miles) [9]. On the other hand, for small sample sizes normality tests often tend to accept the null hypothesis of normality [25]. This could lead to serious problems while conducting t and F-tests as the results become less reliable under significant deviations from normality. This highlights the need to evaluate the performance of normality tests under different degrees of nonnormality and sample sizes to better understand their application and effectiveness in practical scenarios.

The comparison of different normality tests has also been discussed by many authors including Shapiro, Wilk, and Chen [29], Farrell and Rogers-Stewart [7], Xavier, Raimundo, and Anibal [9], Noughabi and Arghami [23], and Torabi, Montazeri, and Grane [35]. In many studies normality tests are compared based on their ability to detect departures from normality using Monte Carlo simulations, by simulating sample data from various alternative distributions. Following this approach, Yazici and Yolacan [38] compared the power of twelve normality tests. Additionally, Yap and Sim [37] compared the power of eight normality tests using data generated from symmetric short-tailed, symmetric long-tailed, and asymmetric distributions as alternatives. Shapiro et.al [29] assessed the sensitivity of nine statistics utilized to evaluate normality using an empirical sampling study under forty-five alternative distributions in twelve families for five sample sizes and analyzed the effect of sample size and parameter misspecification on test performance.

Considering various sample sizes, levels of significance, and four non-normal distributions as alternatives Fiaz et al. [8] studied the performance of twelve normality tests, while Jurgita et al. [17] assessed the performance of 40 normality tests and introduced a novel test based on the N metric approach, specifically for sample sizes exceeding 118. Additionally, Taewoong et al. [34] incorporated both *p*-values and empirical power in their comparative analysis. Stanislaus [32] conducted a comprehensive comparison of 50 normality tests developed between 1900 and 2018, utilizing various symmetric and asymmetric distributions as alternatives. The findings of these studies highlight the need to consider the characteristics of the distributions, along with sample size, when choosing a normality test for statistical analysis.

Although several studies have assessed the performance of normality tests, they mostly focus on extreme deviations, leaving uncertainty about how well these tests perform under mild to moderate non-normality. Only limited studies examine the effectiveness of these tests under slight deviations from normality. This study aims to address this gap by evaluating various normality tests, widely used in real-world applications due to their availability in most statistical software, using simulated non-normal data generated through the Fleishman method. By varying skewness and kurtosis across different sample sizes, this study aims to provide a comprehensive evaluation of normality tests under different degrees of non-normality, offering valuable insights into their size and power.

The remainder of this paper is structured as follows. Sect. "Methods" describes the various normality tests and the simulation setup. Sect."Results " presents the simulation results, focusing on the estimated Type I error rates and power of the various tests. Sect."Discussion" highlights the importance of reliable tests by using illustrative examples. Finally, Sect. "Conclusion and recommendation" provides a discussion of the findings, and Sect. 6 concludes the paper.

## Methods

This section outlines the methodology of all the 13 tests considered in the study which have been selected based on performance in terms of power. Statistical tests for assessing normality can be broadly classified into four categories as those based on regression and correlation (tests based on the ratio of two weighted least square estimates of scales obtained from order statistics), empirical distribution function (tests based on comparison of hypothetical and empirical distribution), measures of moments (tests that detect departure from normality based on sample moments), and chi-square (goodness of fit tests that establish if an observed frequency distribution differs from the theoretical distribution).

Notations used:

**Table:** 

$m$	:	Mean vector whose elements are the expected values of the order statistics of random sample of size *n* from a standard normal distribution
$n$	:	Sample size
$V$	:	Covariance matrix of normal order statistics
${\varvec{\phi}}$	:	CDF of standard normal distribution
${\text{x}}_{\left(\text{i}\right) }$	:	i^th^ the order statistic
$\overline{x }$	:	Sample mean
$s$	:	Sample standard deviation
$\sqrt{{b}_{1}}$	:	Sample skewness
${b}_{2}$	:	Sample kurtosis
${s}_{n}$	:	Classical standard deviation
${J}_{n}$	:	Average absolute deviation from the sample median
${O}_{i}$	:	Observed Frequency of class $i$
${E}_{i}$	:	Expected frequency of class $i$
${p}_{(i)}$	:	CDF at the standardized value of the i^th^ observation
${\widehat{\mu }}_{2}$	:	Second central moment
${\widehat{\mu }}_{3}$	:	Third central moment
${\widehat{\mu }}_{4}$	:	Fourth central moment
${s}_{n}$	:	Standard Deviation
${J}_{n}$	:	Average absolute deviation from sample median

### Regression and correlation-based tests

#### Shapiro Wilk test (SW)

Shapiro and Wilk (1965) [28] test is one of the most powerful regression-based tests of normality. Let $m$ denotes mean vector whose elements are the expected values of the order statistics of a random sample of size $n$ from a standard normal distribution and $V$ is the covariance matrix of those normal order statistics. Then SW test statistic based on $m$ and $V$ is giving by

1 $$\begin{aligned}&W= \frac{{\left[\sum_{i=1}^{n}{a}_{i}{x}_{(i)}\right]}^{2}}{\sum_{i=1}^{n}{\left({x}_{(i)}-\overline{x }\right)}^{2}};\text{ where coefficients }\\&\left({a}_{1}, {a}_{2},\dots..{a}_{n}\right)=\frac{{m}{\prime}{V}^{-1} }{{{(m}{\prime}{V}^{-1}m {V}^{-1})}^\frac{1}{2}} \end{aligned}$$

#### Shapiro Francia test (SF)

In the case of large samples, computation of $V$^−1^ in the Shapiro–Wilk test is extremely time-consuming. To overcome this Shapiro and Francia [30] modified the Shapiro–Wilk test as

2 $${W}_{SF}= \frac{\sum_{i=1}^{n}\left({x}_{\left(i\right)}-\overline{x }\right)({m}_{i}-\stackrel{-}{m)}}{\sqrt{\sum_{i=1}^{n}{\left({x}_{i}-\overline{x }\right)}^{2}\sum_{i=1}^{n}{\left({m}_{i}-\overline{m }\right)}^{2}}}$$

where ${m}_{i}$ denotes the i^th^ element of the mean vector $m$. The value of the above test statistics ranges between 0 and 1 with values close to unity indicating normality.

### Empirical distribution based tests

#### Lilliefors’ test (LF)

When the distribution is not completely specified, the use of the Kolmogorov Smirnov test statistic is unsuitable, as the size tends to be smaller than those specified in the standard table. For such cases, Lilliefors [20] modified the KS test statistic for the test of normality as follows:

3 $$\text{D}=\text{ max }\{\text{D}^+,\text{D}^-\}$$

where $$\mathrm D^+=\underset{i=1,2,...n}{\mathrm{max}}\left\{\frac{\mathrm i}{\mathrm n}-{\mathrm p}_{\left(\mathrm i\right)}\right\}$$, $$\mathrm D^-=\underset{i=1,2,...n}{\mathrm{max}}\left\{{\mathrm p}_{\left(\mathrm i\right)}-\frac{\mathrm i-1}{\mathrm n}\right\}$$ and $$P_{\left(i\right)}=\mathrm\phi\left[\frac{{\mathrm x}_{\left(\mathrm i\right)}-\overline{\mathrm x}}{\mathrm s}\right]$$.

#### Cramer Von Mises Test (CVM)

The Cramer-Von-Mises test is a non-parametric test whose modified test statistic for *p*-value computation is given by

4 $$Z=W\left(1+\frac{0\cdot 5}{n}\right)$$

where $$W=\frac1{12n}+\sum_{i=1}^n\left(p_{(i)}-\frac{2i-1}{2n}\right)^2\;\mathrm{and}\;$$$${p}_{(i)}={\varvec{\phi}}\left[\frac{{x}_{\left(i\right)}-\overline{x}}{s }\right]$$.

#### Anderson Darling Test (AD)

Anderson darling test is a goodness of fit test which is based on empirical distribution. It gives more weights to the tails of the distribution than K-S test. The proposed test statistic is

5 $$\begin{aligned}\text{AD}=&-n-\frac{1}{n} \sum_{i=1}^{n}\left[2i-1\right] [\text{ln}\left({p}_{\left(i\right)}\right)\\&+\text{ln} (1- {p}_{\left(n-i+1\right)})]\end{aligned}$$

### Moment based tests

#### Jarque Bera test (JB)

The Jarque Bera test suggested by Jarque and Bera [15] based on sample skewness and kurtosis is given by

6 $$B= \frac{n}{6} {\left(\sqrt{{b}_{1}}\right)}^{2}+ \frac{n}{24}{ \left({b}_{2}-3\right)}^{2}$$

where $\sqrt{{b}_{1}}=\frac{{\widehat{\mu }}_{3}}{{{\widehat{\mu }}_{2}}^\frac{3}{2}}$ is the sample skewness and ${b}_{2}= \frac{{\widehat{\mu }}_{4}}{{{\widehat{\mu }}_{2}}^{2}}$ is the sample kurtosis respectively.

#### Adjusted Jarque Bera Test (AJB)

Urzua (1996) [36] extended the Jarque Bera test for its enhanced performance, especially in the case of small and medium samples, by considering the exact means and variances of the standardized third and fourth moments rather than their asymptotic counterparts. The Adjusted Jarque Bera test statistic is given by

7 $$\mathrm{AJB} = \frac{\left(\sqrt{b_{1}}\right)^{2}}{\mathrm{Var}\left(\sqrt{b_{1}}\right)} + \frac{\left(b_{2}-\mathrm{E}\left(b_{2}\right)\right)^{2}}{\mathrm{Var}\left(b_{2}\right)}$$

where

$$\mathrm{Var}\left(\sqrt{b_{1}}\right) = \frac{6(n-2)}{(n+1)(n+3)},\; \mathrm{Var}\left(b_{2}\right) = \frac{24n(n-2)(n-3)}{(n+1)^{2}(n+3)(n+5)},\; \mathrm{E}\left(b_{2}\right) = \frac{3(n-1)}{n+1}$$

#### D’ Agostino Skewness Test (DAS)

D’Agostino Skewness Test introduced by D’Agostino [4] utilizes the normalized transformation of the skewness statistic (skew) for sample sizes larger than 8, providing a robust measure of skewness. D’Agostino Skewness Test statistic has the following form,

8 $${z}_{s}=b*\text{ln}(u+\sqrt{{u}^{2}+1})$$

where $${z}_{s}$$ approximately follows standard normal distribution and $$b=\frac{1}{\sqrt{\text{ln}\left(w\right)}};{ w}^{2}= -1+\sqrt{2\left(c-1\right)}$$$$u=a*skew*\sqrt{\frac{\left(n+1\right)\left(n+3\right)}{6\left(n-2\right)}};a=\sqrt{\frac{{w}^{2 }-1}{2}} and$$$$\,skew=\frac{\frac{1}{n}\sum_{i=1}^{n}{\left({x}_{i}-\overline{x }\right)}^{3}}{{\left(\frac{1}{n}\sum_{i=1}^{n}{\left({x}_{i}-\overline{x }\right)}^{3}\right)}^\frac{3}{2}} c=\frac{3\left({n}^{2}-27n-70\right)\left(n+1\right)\left(n+3\right)}{\left(n-2\right)\left(n+5\right)\left(n+7\right)\left(n+9\right)}$$

#### D’ Agostino Kurtosis test (DAK)

The DAK test is based on the assumption that for normally distributed data the test statistic ${z}_{k}$ has a standard normal distribution

9 $${z}_{k}=\frac{1-r-{v}^\frac{1}{3}}{\sqrt{r}}\sim N\left(\text{0,1}\right)$$

where $$\;r=\frac2{9\text{f}}\;\mathrm{and}\;\text{v}=\frac{1-\frac2{\text{f}}}{1+\text{g}}\;\;f=6+\frac8e\left(\frac2e+\sqrt{1+\frac4{e^2}}\right);$$$$e=\frac{6\left(n^2-5n+2\right)}{\left(n+7\right)\left(n+9\right)}\sqrt{\frac{6\left(n+3\right)\left(n+5\right)}{n\left(n-2\right)\left(n-3\right)}}; g=d\ast\left(kurt-\frac{3\left(n-1\right)}{\left(n+1\right)}\right)\sqrt{\frac2{f-4}};$$$$d=\sqrt{\frac{\left(n+1\right)^2\left(n+3\right)\left(n+5\right)}{24n\left(n-2\right)\left(n-3\right)}}$$$$and\;kurt=\frac{\frac1n\sum_{i=1}^n\left(x_i-\overline x\right)^4}{\left(\frac1n\sum_{i=1}^n\left(x_i-\overline x\right)^3\right)^\frac32}$$

#### D’ Agostino Pearson Test (DAP)

The test proposed by D'Agostino and Pearson [5] combines normalized transformations for skewness and kurtosis. This combination ensures that the resulting test statistic follows a chi-square distribution with 2 degrees of freedom. However, for the approximation to be reliable, the sample size must be at least 20.

10 $${{K=z}_{s}}^{2 }+ {{z}_{k}}^{2}$$

where ${z}_{s}$ and ${z}_{k}$ are defined as in the Eq. (8) and (9)

#### Gel Miao Gastwirth Test (GMG)

Gel, Miao, and Gastwirth [12] proposed a directed test of normality for detecting heavy-tailed alternatives. This test compares two variance estimators for normal data: the classical standard deviation $\left(s_n\right)$ and the more robust average absolute deviation from the sample median $\left(J_n\right)$.

11 $$R=\frac{{s}_{n}}{{J}_{n}}$$

where ${J}_{n}=\frac{\sqrt{\frac{\pi }{2}}}{n} \sum_{i=1}^{n}\left|{x}_{i}-median\left({x}_{1},\dots \dots {x}_{n}\right)\right|$. For normally distributed data the value of test statistic is close to 1.

#### Robust Jarque Bera Test (RJB)

A modification of the Jarque–Bera test introduced by Gel and Gastwirth [11] known as RJB test uses an estimate of dispersion that is robust to outliers in skewness and kurtosis instead of the variance used in sample moments. The robust form of the RJB test statistic is given by:

12 $$RJB= \frac{n}{6} {\left( \frac{{\widehat{\mu }}_{3}}{{{J}_{n}}^{3}}\right)}^{2}+ \frac{n}{64}{ \left( \frac{{\widehat{\mu }}_{4}}{{{J}_{n}}^{4}}-3\right)}^{2}$$

The above tests statistic asymptotically follows ${\chi }^{2}$ distribution with 2 degrees of freedom.

### Chi square based test

#### Pearson’s Chi-square test (PCHI)

This goodness-of-fit test, introduced by Pearson [26], compares the observed frequencies to the expected frequencies under a hypothesized distribution.

13 $$P=\sum_{i=1}^{n}\frac{{\left({O}_{i}-{E}_{i}\right)}^{2}}{{E}_{i}}$$

A detailed note on the rejection region of the considered test can be obtained by referring Ag-Yi and Aidoo [1], Romao et.al [27] and Yazici & Yolacan [38].

### Simulation Setup

To evaluate the performance of 13 normality tests based on their estimated Type I error rate and statistical power, a Monte Carlo simulation study was conducted. The effect of sample size on test performance was also examined by considering small, moderate, and large samples, with sizes of 20, 30, 40, 50, 80, 100, and 200. To estimate the Type I error rate, data were generated from normal distributions with varying location and shape parameters specifically, $N(\text{0,1}), N(\text{3,1})$ and $N(\text{0,0.5}).$ Each simulation experiment was replicated 10,000 times. The estimated Type I error rate was computed as the proportion of times the true null hypothesis of normality was rejected out of the 10,000 replications. The nominal significance level was fixed at 5% throughout the experiment. If the estimated Type I error falls within the range of 0.045 to 0.055, it indicates that the test has adequately maintained the nominal significance level. The performance of normality tests available in the *fBasics* and *lawstat* packages in R are considered for evaluation. If the normality test yields a p-value less than 0.05, the null hypothesis of normality is rejected, indicating that the data is not normally distributed. The null and the alternative hypotheses for each of the tests are.

- H_o_: The data follows normal distribution.
- H_1_: The data does not follow normal distribution.

To generate various non-normal distributions the Fleishman’s Power method [10] for non-normal data generation was used. This method being one of the easiest and fastest to execute, generates non-normal data using the first four moments of a random variable without knowing its exact distribution. The R package *detectnorm* was used to generate the non-normal data. The skewness and kurtosis values are varied to generate data with slight to severe deviations from normality as follows.

## Power under slight skewness with fixed kurtosis

To understand how minor deviations from symmetry affect the power of normality tests, data were generated with fixed kurtosis of 3 and Skewness levels set at ± 0.1, ± 0.3 and ± 0.5, representing slight departures from symmetry.

## Power under slight kurtosis variation with fixed symmetry

To understand the effect of kurtosis on the power of the tests while keeping the data symmetric: data were generated with Skewness fixed at 0 and Kurtosis values were varied from 2.5 to 3.5 in increments of 0.2.

## Power under significant deviations in skewness and kurtosis

To assess the robustness of normality tests under significant deviations from the normal distribution, simulations were conducted across a wide range of skewness and kurtosis values. Skewness was classified into three levels: symmetric (skew = 0), moderately skewed (skew = 0.75), and highly skewed (skew = 4) Similarly, kurtosis was varied as platykurtic (kurtosis = 1, 2) representing light tails, mesokurtic (kurtosis = 3) and leptokurtic (kurtosis = 4, 9) indicating heavy tails.

For each selected combination of skewness and kurtosis, datasets of varying sample sizes were repeatedly generated and subjected to the chosen normality tests. Power was then estimated as the proportion of times each normality test correctly rejects the null hypothesis of normality under these non-normal scenarios. The simulations were performed using R software version 4.4.2

## Results

### Simulation results

The performance of the tests were compared based on their size and power values under different scenarios. Power values were computed under scenarios where non-normal data has slight deviations from normality due to skewness or kurtosis alone, as well as substantial deviations involving both skewness and kurtosis. The simulation results are summarized in this section.

#### Size comparison

Evaluating the performance of normality test involves ensuring that it maintains the expected type I error rate under normality. An overly liberal test may falsely reject the true null hypothesis of normality too often, while an overly conservative test may fail to detect true existing deviations. Table 1 provides the empirical size values of the tests under normally distributed data.

**Table 1:** Size of various normality tests at α = 0.05 level of significance

	**N**	**DAP**	**DAS**	**DAK**	**CVM**	**LF**	**SW**	**SF**	**AD**	**PCHI**	**JB**	**AJB**	**RJB**	**GMG**
**N(0,1)**	20	0.0531	0.0456	0.0422	0.0491	0.0452	0.0454	0.0482	0.0468	0.0477	0.021	0.0587	0.0576	0.0647
	30	0.0602	0.0537	0.0496	0.0584	0.0524	0.0555	0.0559	0.0559	0.046	0.0335	0.0623	0.0651	0.0634
	40	0.0556	0.0462	0.0527	0.0468	0.0495	0.0467	0.048	0.0451	0.0578	0.0338	0.0579	0.0583	0.0585
	50	0.062	0.0532	0.0552	0.0536	0.0519	0.0539	0.0562	0.0525	0.049	0.0401	0.0614	0.0652	0.0615
	80	0.051	0.0468	0.0519	0.048	0.0521	0.0473	0.0516	0.0473	0.051	0.0376	0.0516	0.0556	0.0548
	100	0.0555	0.0503	0.0553	0.048	0.0476	0.05	0.0546	0.0489	0.0526	0.0424	0.0572	0.0593	0.0552
	200	0.0528	0.0517	0.0489	0.0496	0.0475	0.0488	0.0483	0.048	0.0516	0.0439	0.0505	0.0511	0.0526
**N(3,1)**	20	0.0561	0.0492	0.0482	0.0517	0.0462	0.0521	0.0536	0.0517	0.0482	0.0254	0.059	0.0599	0.0623
	30	0.0552	0.0458	0.0497	0.0505	0.0493	0.0464	0.0493	0.0485	0.0505	0.0283	0.0552	0.0584	0.0605
	40	0.058	0.0498	0.0543	0.052	0.0476	0.0518	0.0504	0.0492	0.0514	0.034	0.058	0.0613	0.0582
	50	0.0553	0.0487	0.0521	0.0519	0.0544	0.0494	0.0514	0.0519	0.0553	0.037	0.0555	0.0608	0.0573
	80	0.059	0.0534	0.057	0.0494	0.0464	0.0462	0.0552	0.0488	0.0518	0.0472	0.0598	0.0642	0.0574
	100	0.0574	0.0516	0.0556	0.0454	0.0492	0.048	0.0538	0.0452	0.0528	0.0432	0.0584	0.06	0.0578
	200	0.0546	0.0454	0.0584	0.0562	0.0562	0.0512	0.0536	0.0514	0.0562	0.0432	0.0518	0.0498	0.0506
**N(0,0.5)**	20	0.0516	0.049	0.0466	0.0476	0.0468	0.0484	0.0522	0.048	0.046	0.0232	0.0582	0.0608	0.0668
	30	0.0576	0.0482	0.053	0.0404	0.0446	0.0488	0.0516	0.0432	0.0456	0.0304	0.0612	0.0596	0.0582
	40	0.061	0.0532	0.0558	0.0516	0.05	0.0506	0.0544	0.0512	0.0604	0.0374	0.0588	0.0656	0.0636
	50	0.053	0.0468	0.053	0.0452	0.048	0.0456	0.05	0.046	0.056	0.0346	0.0546	0.0576	0.0532
	80	0.056	0.0476	0.055	0.0492	0.05	0.0458	0.0512	0.048	0.0474	0.041	0.0502	0.0562	0.0548
	100	0.0564	0.0482	0.0508	0.0526	0.0506	0.05	0.0472	0.05	0.0502	0.0414	0.0532	0.0598	0.0564
	200	0.0572	0.0536	0.0554	0.0514	0.0496	0.0518	0.0556	0.0526	0.0522	0.0472	0.0548	0.057	0.054

The results from Table 1 suggest that under N (0,1) tests like DAS, DAK, CVM, LF, SW, SF, PCHI and AD maintain the nominal size with better accuracy compared to other tests. At smaller sample sizes (e.g., *n* = 20 or *n* = 30), many tests show greater variability in error rates, with some deviating noticeably from the nominal significance level of 0.05. Particularly, the JB test is notably conservative, showing too small type 1 error rate than the nominal size across all the sample sizes. On the other hand, the AJB, RJB, GMG, and DAP tests are liberal in maintaining size up to a sample size of 100, with the RJB showing deviations by substantial margins. As the sample size increases (*n* = 100 and *n* = 200), the sizes of most tests begin to stabilize and align more closely with the nominal level. The same results hold for N (3,1) and N (0,0.5) for all the tests, except for DAP, which becomes liberal under the varied parameters.

### Power comparison

The effectiveness of a normality test depends on its ability to identify variations in skewness and kurtosis. A highly sensitive test can detect even slight departures from normality. To assess the sensitivity of the tests, their power values are analyzed using non-normal data. The power estimation is based on a simulation framework in which non-normal data is generated using the Fleishman transformation method. This method enables precise control over skewness and kurtosis levels through polynomial transformations.

#### Effect of slight deviations in skewness while controlling kurtosis

To evaluate the impact of skewness on normality tests, non-normal data with a fixed kurtosis of 3 and slight deviations in skewness from zero were generated using the Fleishman method. Skewness values of ± 0.1, ± 0.3, and ± 0.5 were considered. Due to symmetry, power values for both positive and negative skewness are closely aligned, and hence, only the power values for positive skewness are summarized in Table 2.

**Table 2:** Power values of normality test under varying skewness levels with fixed kurtosis

Skew	N	DAP	DAS	DAK	CVM	LF	SW	SF	AD	PCHI	JB	AJB	RJB	GMG
0.1	20	0.2582	0.2232	0.1958	0.1756	0.1424	0.2016	0.2492	0.1902	0.0952	0.1808	0.2888	0.292	0.2908
	30	0.3152	0.264	0.2628	0.2276	0.17	0.269	0.3192	0.2432	0.1066	0.2702	0.3608	0.3848	0.3796
	40	0.3782	0.302	0.3342	0.2764	0.2132	0.33	0.3964	0.3	0.1384	0.3508	0.4356	0.4684	0.4628
	50	0.4346	0.324	0.3974	0.3306	0.2434	0.3962	0.466	0.3598	0.1428	0.4268	0.493	0.5326	0.5278
	80	0.5552	0.3646	0.5448	0.4456	0.3326	0.5226	0.5982	0.4784	0.177	0.5754	0.6328	0.6696	0.6742
	100	0.628	0.3864	0.6288	0.5202	0.4096	0.6054	0.6764	0.558	0.2054	0.6588	0.7068	0.7416	0.7564
	200	0.855	0.4502	0.8808	0.7962	0.6536	0.8652	0.8978	0.834	0.3624	0.8898	0.9064	0.9308	0.9396
0.3	20	0.248	0.2208	0.1966	0.1736	0.1434	0.2034	0.2424	0.1892	0.097	0.1798	0.276	0.2898	0.2884
	30	0.3116	0.2676	0.2622	0.2292	0.1762	0.266	0.3186	0.2474	0.1176	0.2646	0.3534	0.3782	0.3676
	40	0.3844	0.3186	0.3284	0.2818	0.2188	0.3442	0.4048	0.31	0.141	0.3584	0.4334	0.4672	0.4464
	50	0.426	0.3322	0.3856	0.3114	0.2376	0.376	0.4462	0.3362	0.1406	0.4204	0.4848	0.5192	0.5064
	80	0.5736	0.401	0.5558	0.4598	0.3628	0.5462	0.6166	0.4938	0.1944	0.5948	0.6448	0.6816	0.6774
	100	0.6356	0.424	0.6278	0.5338	0.4132	0.6188	0.6818	0.5762	0.2136	0.6654	0.707	0.7428	0.7466
	200	0.8654	0.5076	0.8796	0.8074	0.6774	0.8724	0.9024	0.8424	0.3784	0.8966	0.912	0.9342	0.9386
0.5	20	0.257	0.233	0.1936	0.188	0.1558	0.216	0.2534	0.2018	0.1068	0.1792	0.2814	0.2924	0.2734
	30	0.3256	0.2836	0.2678	0.2406	0.193	0.2824	0.333	0.26	0.128	0.2824	0.3646	0.3862	0.3652
	40	0.3952	0.338	0.331	0.2962	0.2298	0.357	0.408	0.3242	0.1552	0.3702	0.4396	0.4678	0.441
	50	0.4586	0.3766	0.404	0.3576	0.276	0.424	0.4826	0.3892	0.164	0.443	0.5154	0.5468	0.5216
	80	0.586	0.4568	0.5384	0.478	0.3688	0.5702	0.6284	0.514	0.2026	0.5998	0.6446	0.6774	0.6564
	100	0.6556	0.4892	0.623	0.5582	0.4488	0.64	0.7004	0.5954	0.2292	0.679	0.7178	0.7538	0.7348
	200	0.8904	0.633	0.8748	0.8338	0.7122	0.8946	0.9196	0.862	0.3988	0.911	0.9242	0.939	0.9332

The findings from the Table 2 indicate that, for a given sample size, an increase in skewness does not result in a very significant rise in power values. As a result, the tests result in a consistent pattern of performance despite the variation in skewness. For small sample sizes (e.g., *n* = 20 and 30), most tests exhibit relatively low power, especially under mild skewness.

Regardless of sample size, RJB, followed by GMG and AJB, shows the higher power among the tests considered, highlighting their ability to detect slight deviations from normality in terms of skewness, even in small samples. In contrast, the PCHI test consistently shows the lowest power. For instance, when skewness is 0.1 and *n* = 200, the power of the PCHI test is only 0.3624, whereas most other tests, except DAS and LF, exhibit power values around 0.8. Additionally, CVM, LF, and DAS (particularly in n > 50) also exhibit lower power values, making them more suitable for scenarios where slight deviations from normality are acceptable.

However, as the sample size grows to 100 and beyond, the tests become more sensitive to departures from normality, with several tests such as AJB, RJB, GMG, and SW achieving power values above 0.9 when *n* = 200.

#### Effect of slight deviations in kurtosis while controlling skewness

**Fig. 1:**
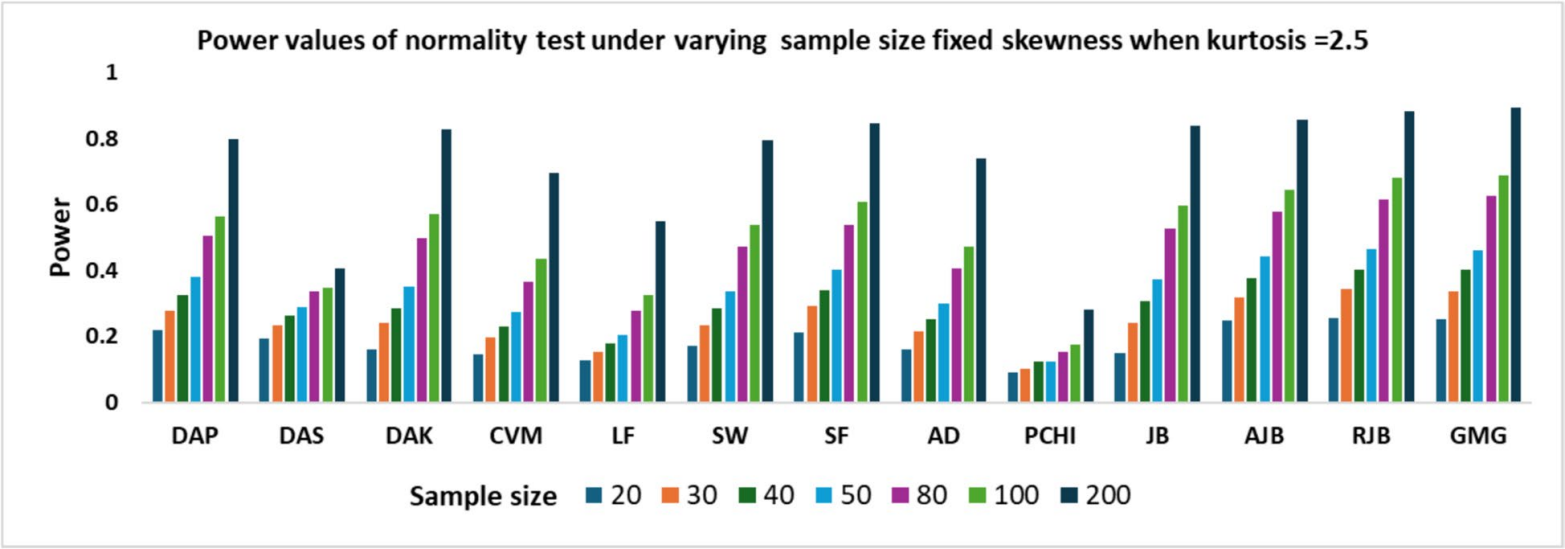
Power values of normality test under varying sample size fixed skewness when kurtosis = 2.5

**Fig. 2:**
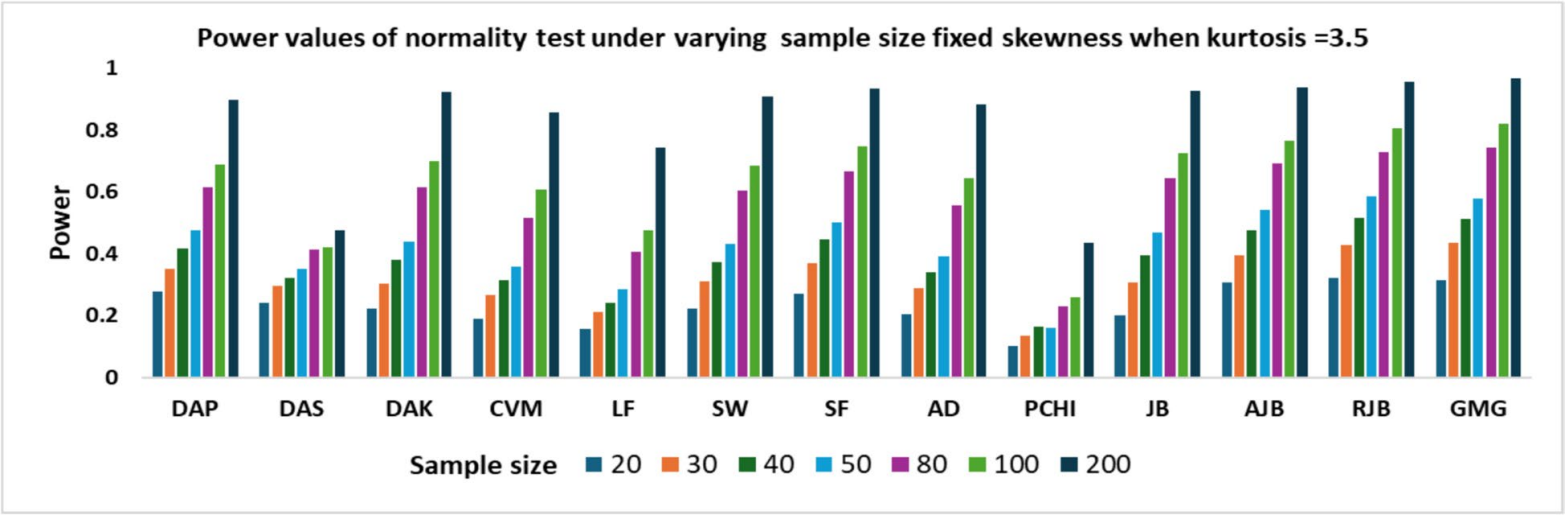
Power values of normality test under varying sample size fixed skewness when kurtosis = 3.5

The impact of kurtosis was analyzed by simulating symmetric (skewness = 0) non-normal observations using the Fleishman method with kurtosis values ranging from 2.5 to 3.5 in increments of 0.2. The power values for kurtosis 2.7 to 3.3 are summarized in Table 3 and power values for kurtosis 2.5 and 3.5 are given in Figs. 1 and 2.

**Table 3:** Power values of normality test under varying kurtosis levels with fixed skewness

Kurt	N	DAP	DAS	DAK	CVM	LF	SW	SF	AD	PCHI	JB	AJB	RJB	GMG
2.7	20	0.23	0.2066	0.1768	0.157	0.1308	0.1876	0.2264	0.1726	0.0954	0.1572	0.2622	0.2698	0.2708
	30	0.2816	0.2346	0.2352	0.2006	0.1584	0.238	0.2932	0.216	0.1008	0.238	0.3264	0.3496	0.342
	40	0.3526	0.274	0.3064	0.2538	0.192	0.3068	0.3664	0.2752	0.137	0.323	0.397	0.4328	0.4224
	50	0.4012	0.302	0.37	0.2818	0.2188	0.3626	0.4278	0.3106	0.1298	0.3918	0.4644	0.4904	0.481
	80	0.5116	0.3428	0.5094	0.3886	0.288	0.4854	0.5548	0.4334	0.1566	0.5362	0.59	0.629	0.6374
	100	0.586	0.3612	0.5906	0.4674	0.362	0.5702	0.6414	0.5192	0.187	0.6268	0.6706	0.7098	0.7168
	200	0.8252	0.4166	0.859	0.7362	0.5952	0.8236	0.8662	0.7764	0.304	0.8622	0.88	0.9044	0.9196
2.9	20	0.2398	0.2134	0.1882	0.1706	0.1378	0.194	0.2352	0.182	0.0928	0.1694	0.2668	0.2786	0.2786
	30	0.3	0.2522	0.2552	0.214	0.1668	0.252	0.3106	0.2252	0.1134	0.258	0.3448	0.367	0.3568
	40	0.3698	0.2904	0.3326	0.261	0.209	0.3194	0.3908	0.285	0.1426	0.3494	0.4274	0.459	0.4516
	50	0.4246	0.3184	0.3864	0.304	0.2386	0.3772	0.4426	0.339	0.1432	0.4168	0.4866	0.5214	0.516
	80	0.5428	0.3594	0.5446	0.429	0.3168	0.5138	0.5856	0.4666	0.1722	0.5666	0.6204	0.6604	0.669
	100	0.6254	0.3896	0.6282	0.5018	0.3864	0.6018	0.6708	0.55	0.1978	0.6584	0.6972	0.7374	0.7528
	200	0.8394	0.436	0.8652	0.7732	0.63	0.8408	0.8802	0.8064	0.3428	0.8736	0.8854	0.9166	0.9358
3.1	20	0.25	0.217	0.1968	0.175	0.1398	0.1958	0.2422	0.1888	0.0968	0.1762	0.2806	0.2902	0.29
	30	0.3226	0.2644	0.2736	0.2258	0.1764	0.2726	0.3278	0.2464	0.1132	0.279	0.3706	0.395	0.3906
	40	0.3796	0.3012	0.3348	0.2792	0.2124	0.339	0.4022	0.3074	0.1412	0.355	0.4346	0.4696	0.4678
	50	0.423	0.3086	0.395	0.311	0.2354	0.3786	0.4532	0.3448	0.1442	0.4172	0.4858	0.5214	0.5226
	80	0.5632	0.3754	0.5604	0.456	0.3408	0.5398	0.6152	0.4868	0.1868	0.589	0.6426	0.6892	0.6934
	100	0.6402	0.3878	0.6456	0.5358	0.4098	0.6242	0.6962	0.573	0.2276	0.6772	0.7208	0.7598	0.7712
	200	0.8698	0.4574	0.8948	0.8044	0.6828	0.8746	0.9096	0.8378	0.3748	0.9038	0.9178	0.9398	0.9468
3.3	20	0.258	0.23	0.2102	0.1866	0.1514	0.2084	0.2548	0.1974	0.095	0.1872	0.2912	0.3022	0.3046
	30	0.335	0.2852	0.286	0.2374	0.1842	0.2888	0.344	0.2644	0.1206	0.2866	0.3802	0.4028	0.4026
	40	0.3996	0.3164	0.3588	0.2904	0.228	0.3506	0.4118	0.3172	0.1542	0.3746	0.4544	0.4854	0.4794
	50	0.4616	0.3476	0.437	0.3512	0.2726	0.4298	0.497	0.3852	0.1584	0.4602	0.5308	0.5664	0.567
	80	0.5928	0.388	0.5988	0.4746	0.372	0.5696	0.6448	0.5144	0.1942	0.6242	0.6712	0.7098	0.719
	100	0.6556	0.4138	0.6734	0.5678	0.4468	0.643	0.7166	0.606	0.2456	0.6934	0.7342	0.777	0.7928
	200	0.89	0.4666	0.9146	0.8432	0.7122	0.897	0.9258	0.8716	0.4038	0.9186	0.9312	0.9508	0.9602

From Table 3, it is evident that for a given sample size, the power values of the test increase with the increase in kurtosis values. The power patterns remain consistent across different kurtosis values. For sample sizes up to 50, the RJB demonstrates the highest power, followed closely by GMG and AJB. However, for n > 50, GMG surpasses RJB in performance. Conversely, PCHI consistently exhibits the lowest power, regardless of sample size and kurtosis values. Additionally, the LF, CVM, and DAS tests also show relatively lower power across all conditions. As the sample size increases from 20 to 200, the power of all tests consistently improves across all levels of kurtosis. This indicates that larger samples enhance the sensitivity of the tests, allowing them to more effectively detect deviations from normality. From Figs. 1 and 2 shows that when kurtosis is fixed at 2.5, the power of normality tests increases with sample size. In small samples (*n* = 20, 30), most tests have low power, but DAS, SW, AJB, RJB, and GMG perform relatively better. As the sample size increases to moderate levels (*n* = 50, 80), the power of tests like RJB, GMG, and AJB improves significantly. At large sample sizes (*n* = 100, 200), these tests consistently show high power, exceeding 0.8. However, tests such as DAK, LF, and PCHI remain weak across all sample sizes, even under moderate kurtosis. A similar pattern is observed when kurtosis is increased to 3.5, with all tests showing higher power compared to when kurtosis is 2.5.

#### Effect of significant deviations from normality in terms of both skewness and kurtosis

An effective normality test should not only detect slight deviations from normality but also reliably identify significant departures. Datasets with varying skewness and kurtosis were generated to understand the performance of normality tests under significant departures from normality. The datasets were categorized based on skewness as symmetric (skew = 0), moderately skewed (skew = 0.75), and highly skewed (skew = 4). Further, based on kurtosis, they were classified as platykurtic (kurt = 1,2), mesokurtic (kurt = 3), or leptokurtic (kurt = 4,9), resulting in various combinations of skewness and kurtosis. By symmetry, the results of positive skewness are consistent with that of the negative, and hence, tables for only positive skewness are presented. The power values of the normality tests under moderately skewed distribution for different values of kurtosis are presented in Table 4.

**Table 4:** Power of test under moderate skewness and varying kurtosis levels

Moderately Skewed (Skew = 0.75)														
n	Kurt	**DAP**	**DAS**	**DAK**	**CVM**	**LF**	**SW**	**SF**	**AD**	**PCHI**	**JB**	**AJB**	**RJB**	**GMG**
20	1	0.1812	0.195	0.11	0.1442	0.1192	0.1826	0.1848	0.1584	0.0872	0.1122	0.183	0.181	0.1328
	2	0.2236	0.219	0.1498	0.1654	0.134	0.1984	0.2206	0.1786	0.0912	0.1528	0.2394	0.2394	0.2048
	3	0.249	0.2436	0.181	0.194	0.1552	0.2196	0.2556	0 [2]	0.1086	0.1784	0.2716	0.2842	0.271
	4	0.3028	0.281	0.2358	0.245	0.1936	0.2702	0.3098	0.2576	0.1274	0.2234	0.3346	0.3444	0.334
	9	0.4436	0.3988	0.3812	0.3998	0.336	0.4114	0.4678	0.4116	0.2276	0.3604	0.4844	0.5224	0.537
30	1	0.244	0.2752	0.1436	0.1922	0.1596	0.2602	0.2572	0.2116	0.1012	0.182	0.2438	0.2406	0.149
	2	0.2858	0.2908	0.194	0.2112	0.1774	0.2616	0.2876	0.2292	0.1112	0.2338	0.3042	0.3134	0.2526
	3	0.3392	0.32	0.256	0.2578	0.207	0.3052	0.343	0.2828	0.1308	0.2852	0.3706	0.385	0.3498
	4	0.3864	0.3508	0.3098	0.3042	0.2432	0.3448	0.4034	0.326	0.1476	0.3392	0.4286	0.4534	0.4306
	9	0.5646	0.4662	0.5198	0.5472	0.448	0.5484	0.6156	0.5642	0.311	0.5252	0.6206	0.6724	0.6936
40	1	0.306	0.3602	0.1618	0.2454	0.1926	0.3402	0.3304	0.2792	0.1256	0.2464	0.3076	0.3062	0.1742
	2	0.3648	0.3678	0.251	0.2726	0.2126	0.3366	0.3702	0.2964	0.1422	0.322	0.3886	0.3964	0.3088
	3	0.4088	0.39	0.3196	0.3364	0.2562	0.39	0.4328	0.358	0.1784	0.3726	0.447	0.4698	0.417
	4	0.4604	0.4004	0.3874	0.3878	0.309	0.4328	0.4832	0.408	0.2052	0.4244	0.5092	0.537	0.5114
	9	0.681	0.5348	0.6322	0.656	0.5602	0.671	0.729	0.6722	0.4072	0.6566	0.7354	0.7876	0.8056
50	1	0.3704	0.4516	0.192	0.3024	0.2364	0.4246	0.412	0.34	0.1408	0.319	0.3714	0.3712	0.1888
	2	0.4236	0.4266	0.2816	0.3326	0.2664	0.419	0.4442	0.3688	0.1406	0.3884	0.4478	0.4584	0.3628
	3	0.4742	0.4422	0.3712	0.3786	0.3002	0.4512	0.4978	0.41	0.1718	0.4532	0.5158	0.5436	0.4854
	4	0.7412	0.5626	0.7192	0.7404	0.641	0.7518	0.8022	0.7566	0.4538	0.741	0.8014	0.8488	0.8676
	9	0.741	0.5698	0.7246	0.7496	0.6486	0.7532	0.8054	0.7656	0.461	0.7478	0.8048	0.8512	0.8702
80	1	0.5432	0.6522	0.249	0.4446	0.362	0.6146	0.5948	0.508	0.1844	0.5094	0.5476	0.5386	0.2318
	2	0.569	0.5808	0.3852	0.4544	0.361	0.5652	0.5932	0.4968	0.188	0.555	0.5974	0.6094	0.4558
	3	0.6468	0.5682	0.532	0.5442	0.4422	0.623	0.6734	0.579	0.232	0.6434	0.6878	0.711	0.631
	4	0.6938	0.5666	0.6272	0.6382	0.5228	0.689	0.7388	0.6624	0.2902	0.7016	0.7432	0.7816	0.758
	9	0.8822	0.6446	0.8866	0.9058	0.8314	0.9044	0.9286	0.9126	0.6412	0.8984	0.9232	0.95	0.9654
100	1	0.6358	0.7436	0.2762	0.5388	0.424	0.7072	0.6898	0.5966	0.2274	0.6114	0.6382	0.6212	0.253
	2	0.6502	0.6512	0.4608	0.5324	0.4364	0.6394	0.6696	0.5748	0.2048	0.6438	0.6786	0.6902	0.5254
	3	0.7118	0.6204	0.607	0.6314	0.5068	0.7048	0.7476	0.6606	0.271	0.7198	0.7522	0.7782	0.7046
	4	0.7748	0.6198	0.7206	0.7246	0.606	0.7816	0.8214	0.755	0.3438	0.7914	0.8216	0.855	0.8334
	9	0.9368	0.6772	0.9406	0.951	0.8992	0.952	0.9654	0.9562	0.7426	0.9502	0.9622	0.9776	0.986
200	1	0.9336	0.9624	0.4348	0.8486	0.7248	0.9524	0.9444	0.8938	0.461	0.9284	0.9352	0.9226	0.366
	2	0.8838	0.8688	0.694	0.8132	0.7106	0.884	0.9006	0.8474	0.3884	0.8852	0.8952	0.9026	0.7288
	3	0.9214	0.7978	0.8618	0.8858	0.7946	0.9202	0.9386	0.9002	0.4862	0.9306	0.9392	0.949	0.9082
	4	0.9568	0.7812	0.937	0.9398	0.8648	0.961	0.9724	0.9518	0.603	0.9652	0.971	0.9802	0.9708
	9	0.998	0.7526	0.998	0.9982	0.9944	0.9992	0.9996	0.9996	0.953	0.999	0.9992	0.9996	1

The results from Table 4 indicate that the DAS test outperforms all the other tests when the kurtosis value is around 1. Observe that power of all normality tests increases as the sample size grows, even under moderate skewness and varying levels of kurtosis. For small samples (e.g., *n* = 20 or 30), the tests show limited ability to detect non-normality, especially when kurtosis is low. For values exceeding 1, the RJB test shows superior performance irrespective of the sample size. The performance of RJB is closely followed by that of AJB and SF tests. For very high kurtosis values, the GMG test consistently exhibits superior performance regardless of the sample size, however, its performance shows a clear decline in case of lower kurtosis values. Overall, the PCHI test consistently performs the worst across all sample sizes. Additionally, the LF and DAK tests also show notably poor performance. The performance of the tests under significantly increased values of skewness is summarized in Table 5.

**Table 5:** Power of test under high skewness and varying kurtosis levels

Highly Skewed (Skew = 4)														
**n**	**Kurt**	**DAP**	**DAS**	**DAK**	**CVM**	**LF**	**SW**	**SF**	**AD**	**PCHI**	**JB**	**AJB**	**RJB**	**GMG**
20	1	0.6098	0.7364	0.3384	0.8402	0.7042	0.9304	0.898	0.8824	0.8388	0.4878	0.614	0.608	0.4494
	2	0.659	0.7838	0.3734	0.8664	0.7388	0.9452	0.918	0.9034	0.8678	0.5324	0.6658	0.6606	0.5028
	3	0.708	0.8208	0.4234	0.8974	0.7842	0.9608	0.9404	0.9312	0.8972	0.5934	0.707	0.7044	0.5634
	4	0.6898	0.794	0.4116	0.882	0.7624	0.9556	0.9282	0.9182	0.8878	0.5678	0.6916	0.687	0.5424
	9	0.7406	0.84	0.4558	0.92	0.8128	0.9742	0.9534	0.946	0.9158	0.6248	0.7438	0.7442	0.6052
30	1	0.8062	0.9104	0.4436	0.967	0.9044	0.9948	0.989	0.9816	0.967	0.7458	0.8188	0.7918	0.5642
	2	0.8376	0.9312	0.477	0.971	0.923	0.996	0.9906	0.986	0.976	0.7856	0.8544	0.8312	0.61
	3	0.8778	0.9546	0.542	0.9846	0.9516	0.9986	0.9956	0.9936	0.9854	0.838	0.8914	0.8766	0.687
	4	0.857	0.9382	0.5242	0.977	0.931	0.9966	0.9918	0.9894	0.977	0.8092	0.8664	0.8488	0.6546
	9	0.895	0.9614	0.575	0.9882	0.9586	0.9984	0.997	0.9936	0.9906	0.855	0.9084	0.8938	0.717
40	1	0.923	0.9748	0.5388	0.9938	0.9758	1	0.9988	0.9978	0.9946	0.9024	0.9306	0.9018	0.6478
	2	0.9498	0.9818	0.584	0.9952	0.9844	0.9996	0.9992	0.999	0.9972	0.9338	0.9566	0.9318	0.7066
	3	0.9634	0.9888	0.6472	0.9968	0.9914	0.9998	0.9996	0.999	0.9988	0.9506	0.9708	0.9502	0.7774
	4	0.9546	0.9876	0.6234	0.9976	0.9906	0.9998	0.9998	0.9998	0.9988	0.9436	0.9642	0.9422	0.7542
	9	0.9708	0.9902	0.6754	0.9984	0.9928	1	0.9998	0.9996	0.9994	0.962	0.9732	0.9598	0.8094
50	1	0.9774	0.9938	0.604	0.9992	0.9952	1	1	0.9998	0.9992	0.9704	0.9806	0.9574	0.7096
	2	0.9872	0.9954	0.6662	0.9994	0.9974	1	1	0.9998	0.9988	0.9824	0.9886	0.972	0.7746
	3	0.996	0.9984	0.7444	0.9998	0.999	1	1	1	0.9998	0.9932	0.9958	0.9872	0.8452
	4	0.9932	0.9978	0.6998	0.9998	0.9988	1	1	0.9998	1	0.9906	0.9942	0.9832	0.8236
	9	0.9956	0.9986	0.7602	1	0.999	1	1	1	0.9996	0.9924	0.996	0.9882	0.8622
80	1	1	1	0.7684	1	1	1	1	1	1	1	1	0.9992	0.8396
	2	1	1	0.8228	1	1	1	1	1	1	1	1	0.9998	0.892
	3	1	1	0.885	1	1	1	1	1	1	1	1	0.9998	0.9532
	4	1	1	0.8554	1	1	1	1	1	1	1	1	0.9996	0.9272
	9	1	1	0.9002	1	1	1	1	1	1	1	1	0.9998	0.9534
100	1	1	1	0.8312	1	1	1	1	1	1	1	1	1	0.8862
	2	1	1	0.8986	1	1	1	1	1	1	1	1	1	0.9408
	3	1	1	0.933	1	1	1	1	1	1	1	1	1	0.9722
	4	1	1	0.9198	0.9996	1	1	1	1	1	1	1	1	0.9552
	9	1	1	0.949	0.9994	1	1	1	1	1	1	1	1	0.981
200	1	1	1	0.9814	0.9212	1	1	1	1	1	1	1	1	0.987
	2	1	1	0.9892	0.8288	1	1	1	1	1	1	1	1	0.9956
	3	1	1	0.9954	0.5806	1	1	1	1	1	1	1	1	0.999
	4	1	1	0.994	0.707	1	1	1	1	1	1	1	1	0.9986
	9	1	1	0.9974	0.4824	1	1	1	1	1	1	1	1	0.9996

The results from Table 5 suggest that regardless of the kurtosis values, the SW test closely followed by SF test shows consistently superior performance up to a sample size of 40, after which SF test outperforms SW test. For sample size exceeding 50, the power of most of the tests converges to 1 showing exceptional power in detecting deviations from normality. As the sample size increases, the power of most normality tests improves markedly, especially under high skewness conditions. Among the tests compared, the DAK test followed by the GMG and JB tests show significantly lower power values, especially for smaller sample sizes.

Finally, to examine the effect of substantial variation in kurtosis, the power of the test is analyzed by maintaining the symmetry. The power values are presented in Table 6

**Table 6:** Power of test under symmetry and varying kurtosis levels

Symmetric (Skew = 0)														
n	Kurt	**DAP**	**DAS**	**DAK**	**CVM**	**LF**	**SW**	**SF**	**AD**	**PCHI**	**JB**	**AJB**	**RJB**	**GMG**
20	1	0.1366	0.12	0.0976	0.0884	0.0752	0.1016	0.1246	0.0952	0.0622	0.081	0.1566	0.1592	0.1528
	2	0 [2]	0.1754	0.1572	0.1314	0.1036	0.1562	0.197	0.1444	0.0784	0.1358	0.2268	0.2388	0.2358
	4	0.296	0.2592	0.239	0.2172	0.1758	0.2442	0.2946	0.228	0.109	0.2176	0.3302	0.346	0.3436
	9	0.44	0.385	0.3862	0.3948	0.3354	0.4042	0.467	0.4064	0.226	0.3566	0.4888	0.5294	0.5572
30	1	0.1608	0.136	0.1264	0.0976	0.083	0.1246	0.1532	0.1074	0.0666	0.1226	0.1804	0.1904	0.1774
	2	0.2424	0.2084	0.2004	0.1626	0.1256	0.2002	0.2436	0.1788	0.0922	0 [2]	0.2818	0.2964	0.2898
	4	0.3752	0.3054	0.325	0.2796	0.2148	0.327	0.3894	0.3032	0.135	0.3308	0.4244	0.4514	0.4492
	9	0.5606	0.4476	0.5268	0.54	0.451	0.5392	0.6072	0.5506	0.3138	0.5178	0.618	0.6784	0.7132
40	1	0.1856	0.1554	0.1496	0.1064	0.0862	0.1436	0.1794	0.1184	0.0788	0.158	0.2148	0.2198	0.2024
	2	0.2812	0.224	0.2428	0.1908	0.148	0.2364	0.293	0.2112	0.1084	0.2574	0.3242	0.3486	0.3372
	4	0.4362	0.3406	0.4026	0.3438	0.2708	0.3984	0.4734	0.3762	0.1876	0.4158	0.5004	0.5396	0.5468
	9	0.6646	0.5018	0.6586	0.6608	0.5596	0.6698	0.7336	0.68	0.4062	0.6578	0.7346	0.7892	0.8196
50	1	0.2084	0.166	0.1772	0.12	0.0952	0.17	0.2122	0.1332	0.0694	0.1952	0.2474	0.2612	0.2382
	2	0.3344	0.2554	0.293	0.2148	0.1678	0.2894	0.3508	0.2464	0.1052	0.3198	0.3854	0.4122	0.387
	4	0.4968	0.3718	0.4716	0.4078	0.3104	0.468	0.5324	0.4324	0.1898	0.4956	0.5668	0.612	0.616
	9	0.7362	0.5372	0.735	0.7364	0.6302	0.7478	0.7988	0.7504	0.4438	0.7416	0.8008	0.8498	0.8698

From Table 6, it is evident that for small samples (*n* = 20,30), the RJB test, closely followed by the GMG test, shows superior performance for kurtosis values ranging between 1 and 4. At small sample sizes (*n* = 20 or 30), most tests show low to moderate power, especially when kurtosis is low. However, for highly heavy-tailed distributions, the GMG test outperforms RJB.

**Fig. 3:**
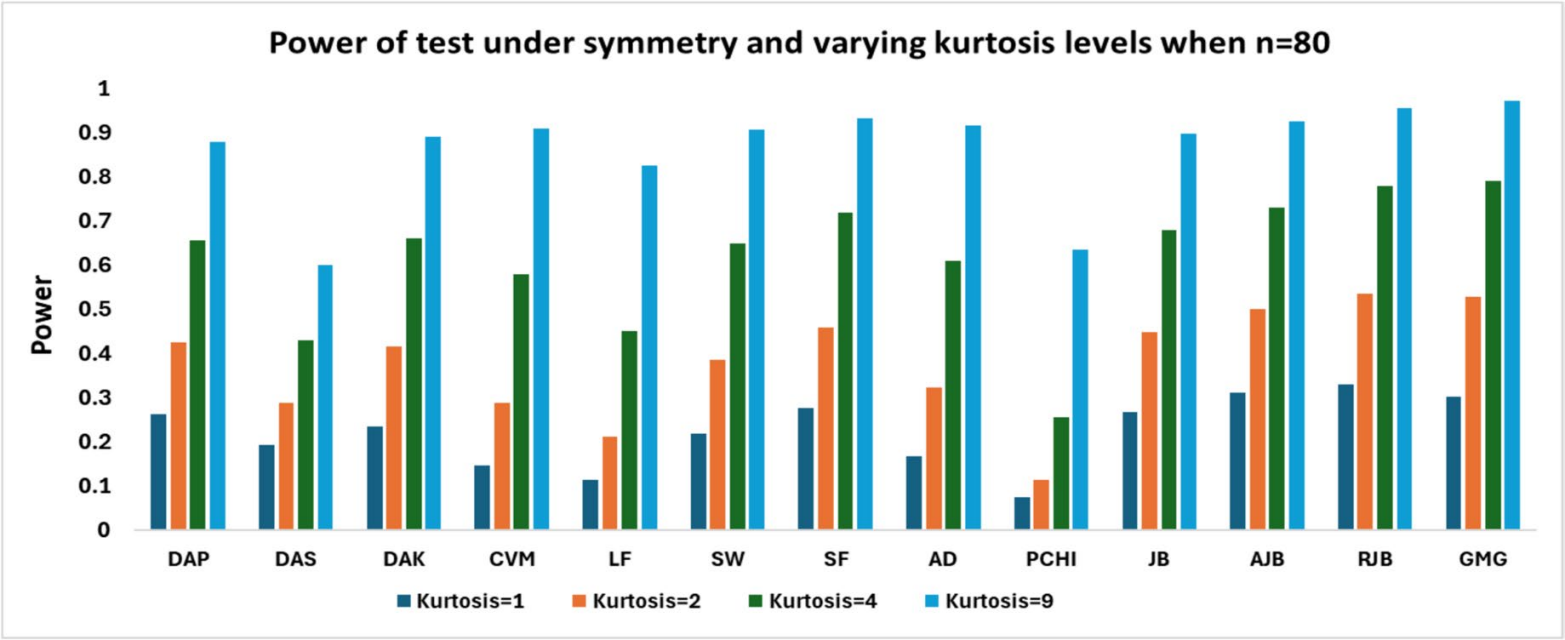
Power of test under symmetry and varying kurtosis levels when *n* = 80

**Fig. 4:**
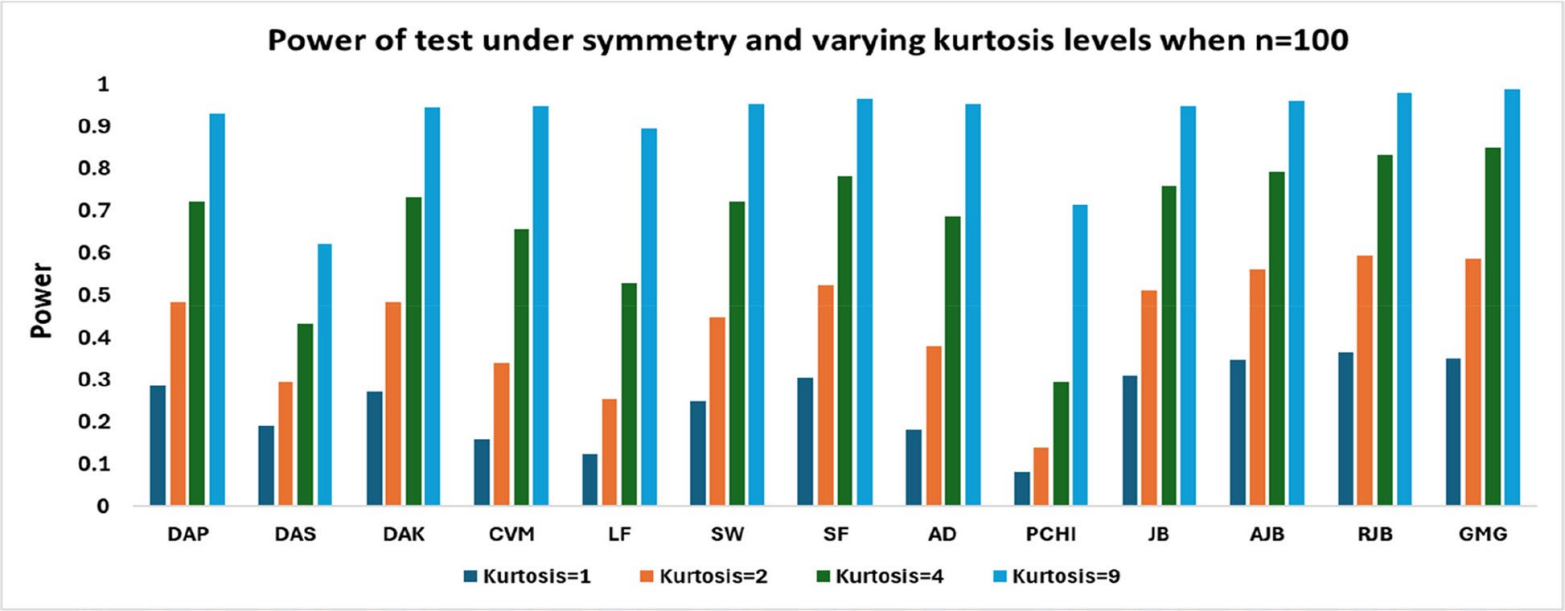
Power of test under symmetry and varying kurtosis levels when *n* = 100

**Fig. 5:**
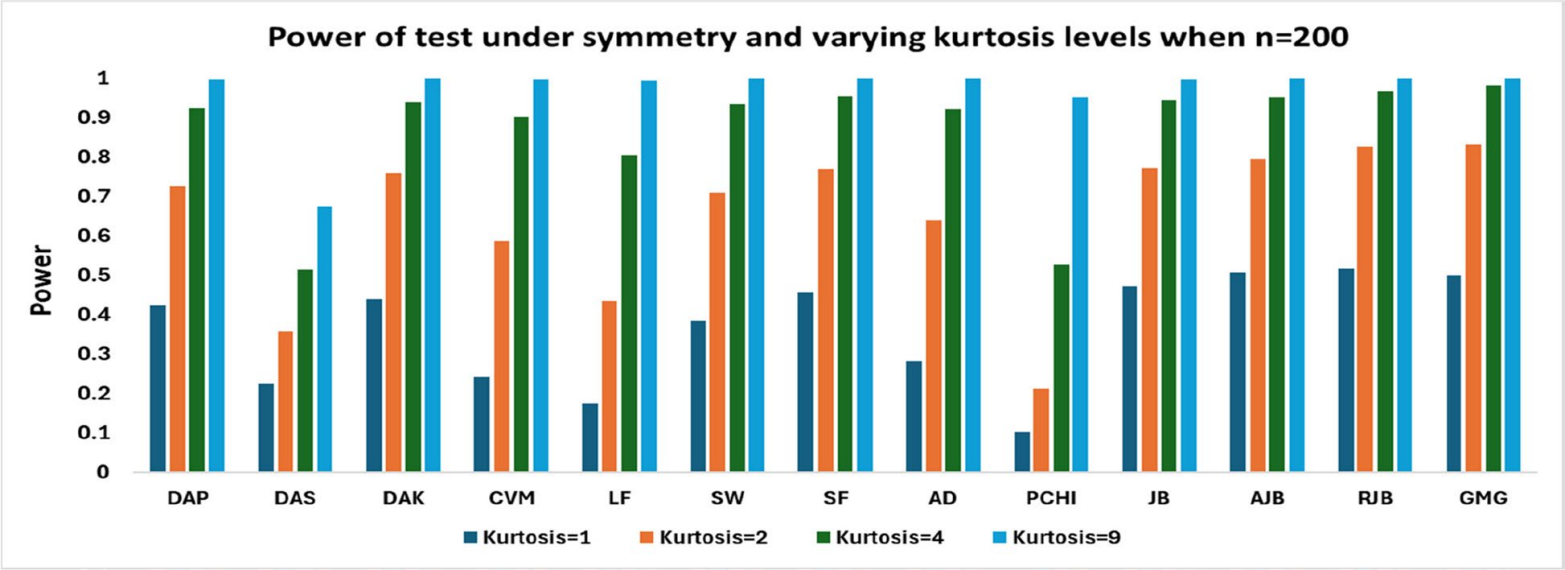
Power of test under symmetry and varying kurtosis levels when *n* = 200

Figs. 3, 4 and 5  shows that with larger sample sizes (n ≥ 80), the power rises sharply across all tests, particularly under high kurtosis. In contrast, for large samples, the RJB test performs better when kurtosis is low (1,2), whereas the GMG exhibits superior performance for higher kurtosis values, with RJB closely following. The PCHI test consistently shows the weakest performance, except in high-kurtosis cases for large samples, where DAS performs the worst. Other tests, such as CVM and LF, also exhibit significantly lower power in comparison.

### Empirical results

This section explores real-life datasets to highlight the critical role of choosing appropriate normality tests. The primary objective here is to demonstrate how different tests can yield varying conclusions about data normality, emphasizing the importance of selecting a reliable and suitable test for analysis.

### Example 1

In 1992, Jolson et al. [16] investigated the occurrence of cerebellar toxicity in 63 leukemia patients treated with high doses of cytarabine. According to the product manufacturer, 25 patients exclusively received the Quad product, and 34 patients received the Upjohn product during a single chemotherapy course. The data provides the total dosage of cytarabine (g/m2). The dataset is available in https://www.sjsu.edu/faculty/gerstman/datasets/toxic.sav

### Remark

Since the Quad manufacturer data represents a moderately positively skewed platykurtic distribution (skewness = 0.787, kurtosis = 2.003) and the Upjohn data follows an almost symmetric platykurtic distribution (skewness = 0.373, kurtosis = 1.741), this study recommends using the RJB test to assess normality in both cases based on the simulation results. From Table 7, it is concluded that the total dosage of cytarabine values follow a normal distribution for both manufacturers.

**Table 7:** Performance of various normality tests for the datasets considered

Tests	*p*- value and Conclusion		
	**Dataset-1**		**Dataset-2**
	**Quad**	**Upjohn**	**Diet A**
SW	< 0.001	0.0013	0.0158
	(Non-Normal)	(Non-Normal)	(Non-Normal)
JB	0.1624	0.2538	0.0379
	(Normal)	(Normal)	(Non-Normal)
AD	< 0.001	< 0.001	0.0424
	(Non-Normal)	(Non-Normal)	(Non-Normal)
KS	0.0133	0.0091	0.6035
	(Non-Normal)	(Non-Normal)	(Normal)
LF	< 0.001	< 0.001	0.1371
	(Non-Normal)	(Non-Normal)	(Normal)
DAS	0.0568	0.2926	0.0137
	(Normal)	(Normal)	(Non-Normal)
DAK	0.3723	0.0200	0.1043
	(Normal)	(Non-Normal)	(Normal)
SF	< 0.001	0.0029	0.0178
	(Non-Normal)	(Non-Normal)	(Non-Normal)
GMG	0.1529	0.7698	0.0421
	(Normal)	(Normal)	(Non-Normal)
RJB	0.1497	0.4008	0.0015
	(Normal)	(Normal)	(Non-Normal)
DAP	0.1096	0.0383	0.0128
	(Normal)	(Non-Normal)	(Non-Normal)
PCHI	< 0.001	< 0.001	0.5494
	(Non-Normal)	(Non-Normal)	(Normal)
CVM	1.7575e-05	6.4089e-05	0.0893
	(Non-Normal)	(Non-Normal)	(Normal)
AJB	0.1127	0.2095	0.0063
	(Normal)	(Normal)	(Non-Normal)

### Example 2

Couturier, D. L. et al. [2] investigated weight loss across three different types of diets (A, B, and C). The study comprised seven variables: gender, age, height, diet type, initial weight, final weight, and weight loss, with a total of 78 observations. Normality was assessed for the weight loss variable across all three types of diets. The dataset is available in https://rdrr.io/rforge/WRS2/man/diet.html

### Remark

Weight loss for diet A demonstrates a moderately positively skewed and leptokurtic distribution (*n* = 24, skewness = 1.2316, kurtosis = 4.6763). Therefore, following the study's recommendation, the RJB test was utilized to assess normality.

The results from Table 7 indicate that the data on weight loss due to Diet A follows a non- normal distribution. For Diet-B and Diet-C, the results of all 13 tests showed *p*-values greater than 0.05 indicating that data on weight loss for Diet-B and Diet-C are normally distributed.

## Discussion

In real-world data, perfect normality is rare, and data distributions often exhibit variations in skewness or kurtosis. Understanding how the normality tests perform in identifying these deviations can help in making the appropriate choice of the tests based on characteristics of the data distribution.

Firstly, the tests were evaluated for size to assess their false rejection rates under true normality. The simulation results highlight the efficiency of DAS, DAK, CVM, LF, SW, SF and AD tests in maintaining the nominal levels of significance even in small sizes. This suggests their ability in ensuring reliable normality assessments.

When the underlying analysis does not require strict normality assumptions, a highly sensitive test may flag even minor deviations from normality, leading to unnecessary concern. The analysis may then require additional transformations, which may complicate interpretation without any substantial benefits. By understanding how normality tests perform in identifying slight deviations from normality, one can select the most suitable test for the intended statistical analysis, ensuring reliable results.

In this study, initially the performance of the normality tests was analyzed under slight deviations from normality by varying skewness with controlled kurtosis and vice versa. The results indicate that compared to other tests, RJB, GMG, and AJB are highly effective in detecting minor deviations from normality (both in terms of skewness and kurtosis), even in small samples. This makes them particularly suitable for testing normality when the underlying analysis requires strict normality assumptions. On the other hand, tests like PCHI, LF, CVM and DAS (particularly for large samples n ≥ 80) fail to detect these slight deviations, making them more suitable for confirming normality in analyses with relaxed normality assumptions.

When the performance of tests was assessed under significant deviations from normality, in most cases, the RJB, GMG, and AJB tests demonstrated the best performance in detecting even major deviations in skewness and kurtosis. However, it is interesting to note that these tests perform well as long as skewness remains near symmetrical, i.e., from slight deviations around 0 to moderate skewness levels. When skewness becomes extreme, the performance of these tests deteriorates significantly, whereas the SW and SF tests exhibit superior performance under these cases.

Across most cases, the PCHI test exhibits poor performance, except when the observations are significantly skewed, where it shows relatively better detection capability. Similarly, tests like CVM and LF demonstrate improved performance primarily under such conditions but remain less effective in detecting slight deviations from normality. Further, the real-life application of these tests based on study’s recommendation highlights the need for choosing the correct normality tests to get reliable conclusions.

The findings of the study highlight that no single test can perform better under all conditions, thus stressing the importance of selecting an appropriate test based on the nature of distributional deviations and sample size. Researchers should carefully consider the distributional characteristics of their data when selecting tests, ensuring that the chosen method aligns with the specific deviations present rather than relying on a one-size-fits-all approach.

### Limitations of the study

It is important to recognize the study's limitations, particularly those associated with the Fleishman method. Since it relies on polynomial transformation to approximate non-normal distributions, the accuracy of the data generated depends on the polynomial coefficients. Also, the skewness and kurtosis values used in the simulation were limited to a few selected combinations only. Future studies could explore a wider range of skewness and kurtosis values and evaluate alternative data generation methods beyond Fleishman’s transformation. While this study evaluates 13 commonly used normality tests, future research can focus on assessing additional tests to provide a more comprehensive comparison.

## Conclusion and recommendation

This study demonstrates that the performance of normality tests is influenced by sample size, skewness, and kurtosis. While most tests effectively detect non-normality in large samples, their power is notably limited in small sample scenarios, particularly under high skewness or kurtosis. Key findings are summarized in Table 8, which offers practical recommendations for selecting the most powerful test under varying distributional conditions.

**Table 8:** Test with highest power under given sample size, skewness and kurtosis combinations

Sample Size	Kurtosis	Moderately Skewed	Highly Skewed	Symmetric
20	1–2	DAS/SW	SW	RJB/GMG
	2–4	RJB/AJB	SW	RJB/GMG
	4–9	GMG/RJB	SW	GMG/RJB
30	1–2	DAS/SW	SW/SF/AD	RJB/GMG
	2–4	RJB/AJB	SW/SF/AD	RJB/GMG
	4–9	GMG/RJB	SW/SF/AD	GMG/RJB
40	1–2	DAS/SW	SW/SF/AD	RJB/GMG
	2–4	RJB/AJB	SW/SF/AD	RJB/GMG
	4–9	GMG/RJB	SW/SF/AD	GMG/RJB
50	1–2	DAS/SW	SW/SF/AD	RJB/GMG
	2–4	RJB/AJB	SW/SF/AD	RJB/GMG
	4–9	GMG/RJB	SW/SF/AD	GMG/RJB
80	1–2	DAS/SW	*(Except DAK & GMG)	RJB/GMG
	2–4	RJB/AJB	*(Except DAK)	RJB/GMG
	4–9	GMG/RJB	*(Except DAK)	GMG/RJB
100	1–2	DAS/SW	*(Except DAK & GMG)	RJB/GMG
	2–4	RJB/AJB	*	RJB/GMG
	4–9	GMG/RJB	*	GMG/RJB
200	1–2	DAS/SW	*(Except CVM)	RJB/GMG
	2–4	RJB/AJB	*(Except CVM)	RJB/GMG
	4–9	GMG/RJB	*(Except CVM)	GMG/RJB

^*^Any of the tests can be used

For moderately skewed data with low kurtosis, the DAS and Shapiro–Wilk (SW) tests perform better across all sample sizes. As kurtosis increases, the RJB and AJB tests are more appropriate, and at high kurtosis levels, GMG and RJB show consistently better performance. For highly skewed data, the SW test remains the most reliable, with SF and AD gaining strength in larger samples. In contrast, tests such as DAK, GMG, and CVM show limited utility under high skewness. For symmetric data, RJB and GMG are robust choices, with GMG preferred at higher kurtosis.

These findings contribute to enhancing statistical practices in public health research by offering practical, evidence-based guidance for selecting appropriate normality tests based on sample size, skewness, and kurtosis. Such informed selection improves the accuracy of statistical analysis and strengthens the credibility and validity of research findings. This is particularly important in public health research, where many commonly used statistical methods rely on the assumption of normality.

## Data Availability

No datasets were generated or analysed during the current study.

## Abbreviations

CDF: Cumulative Distribution Function
SW: Shapiro Wilk test
SF: Shapiro Francia test
LF: Lilliefors’ test
CVM: Cramer Von Mises Test
AD: Anderson Darling Test
JB: Jarque Bera test
AJB: Adjusted Jarque Bera Test
DAS: D’ Agostino Skewness Test
DAK: D’ Agostino Kurtosis test
DAP: D’ Agostino Pearson Test
GMG: Gel Miao Gastwirth Test
RJB: Robust Jarque Bera Test
PCHI: Pearson’s Chi-square test

## References

[1] Ag-Yi D, Aidoo EN. A comparison of normality tests towards convoluted probability distributions. Res Math. 2022;9(1): 2098568.

[2] Couturier DL, Nicholls R, Fernandes M: ANOVA with R: Analysis of the diet dataset. Retrieved online. 2018.

[3] Cramér H. On the composition of elementary errors. Scand Actuar J. 1928;1928(1):141–80.

[4] D’Agostino R. Simple compact portable test of normality: Geary’s test revisited. Psychol Bull. 1970;74:138–40.

[5] D’Agostino R, Pearson ES. Tests for departure from normality: empirical results for the distributions of b₂ and √b₁. Biometrika. 1973;60(3):613–22.

[6] Das KR, Imon AH: A brief review of tests for normality. American Journal of Theoretical and Applied Statistics. 2016, 5 (12).

[7] Farrell PJ, Rogers-Stewart K. A comprehensive study of tests for normality and symmetry: extending the Spiegelhalter test. J Stat Comput Simul. 2006;76(9):803–16.

[8] Fiaz A, Rehan AK: A power comparison of various normality tests. Pakistan Journal of Statistics and Operation Research. 2017.

[9] Field A, Miles J: Discovering statistics using SAS. 2010.

[10] Fleishman AI. A method for simulating non-normal distributions. Psychometrika. 1978;43:521–32.

[11] Gel YR, Gastwirth JL. A robust modification of the Jarque-Bera test of normality. Econ Lett. 2008;99(1):30–2.

[12] Gel YR, Miao W, Gastwirth JL. Robust directed tests of normality against heavy-tailed alternatives. Comput Stat Data Anal. 2007;51(5):2734–46.

[13] Huber PJ. Robust regression: Asymptotics, conjectures, and Monte Carlo. Annals Statist. 1973;1(5):799–821. 10.1214/aos/1176342503.

[14] Jarque CM, Bera AK. Model specification tests: A simultaneous approach. J Econometr. 1982;20:59–82.

[15] Jarque CM, Bera AK. A test for normality of observations and regression residuals. Int Stat Rev Revue Int Stat. 1987;55(2):163–72.

[16] Jolson HM, Bosco L, Bufton MG, Gerstman BB, Rinsler SS, Williams E, Flynn B, Simmons WD, Stadel BV, Faich GA. Clustering of adverse drug events: analysis of risk factors for cerebellar toxicity with high-dose cytarabine. J Natl Cancer Inst. 1992;84(7):500–5. 1545440 10.1093/jnci/84.7.500

[17] Jurgita A, Tomas R, Mindaugas B. An exhaustive power comparison of normality tests. Mathematics. 2021;9(7):788.

[18] Knief U, Forstmeier W. Violating the normality assumption may be the lesser of two evils. Behav Res Methods. 2021;53(6):2576–90. 33963496 10.3758/s13428-021-01587-5 PMC8613103

[19] Koenker RW. Robust methods in econometrics. Econometr Rev. 1982;1:213–90.

[20] Lilliefors HW. On the Kolmogorov-Smirnov test for normality with mean and variance unknown. J Am Stat Assoc. 1967;62(318):399–402.

[21] Lumley T, Diehr P, Emerson S, Chen L. The importance of the normality assumption in large public health data sets. Annu Rev Public Health. 2002;23:151–69. 11910059 10.1146/annurev.publhealth.23.100901.140546

[22] Mises R von: Wahrscheinlichkeitsrechnung und ihre Anwendung in der Statistik und theoretischen Physik. F. Deuticke. 1931.

[23] Noughabi HA, Arghami NR. Monte carlo comparison of seven normality tests. J Stat Comput Simul. 2011;81(8):965–72.

[24] Orcan F. Parametric or non-parametric: skewness to test normality for mean comparison. Int J Assess Tools Educ. 2020;7(2):255–65.

[25] Öztuna D, Elhan AH, Tüccar E. Investigation of four different normality tests in terms of type I error rate and power under different distributions. Turk J Med Sci. 2006;36(3):171–6.

[26] Pearson K: On the criterion that a given system of deviations from the probable in the case of a correlated system of variables is such that it can be reasonably supposed to have arisen from random sampling. Philosophical Magazine. 1900, Series 5, 50 (302): 157–175.

[27] Romão X, Delgado R, Costa A. An empirical power comparison of univariate goodness-of-fit tests for normality. J Stat Comput Simul. 2010;80(5):545–91.

[28] Shapiro SS, Wilk MB. An analysis of variance test for normality (complete samples). Biometrika. 1965;52(3–4):591–611.

[29] Shapiro SS, Wilk MB, Chen HJ. A comparative study of various tests for normality. J Am Stat Assoc. 1968;63(324):1343–72.

[30] Shapiro SS, Francia RS. An approximate analysis of variance test for normality. J Am Stat Assoc. 1972;67(337):215–6.

[31] Shatz I. Assumption-checking rather than (just) testing: the importance of visualization and effect size in statistical diagnostics. Behav Res. 2024;56:826–45. 10.3758/s13428-023-02072-x PMC10830673 36869217

[32] Stanislaus SU: An extensive comparison of 50 univariate goodness-of-fit tests for normality. Austrian J Statistics. 2022. 10.17713/ajs.v51i3.1279

[33] Stephens MA: Tests based on EDF statistics. In: D’Agostino RB, Stephens MA (eds). Goodness-of-fit techniques. Marcel Dekker. 1986, 68: 97–185.

[34] Taewoong U, Seongbaek Y. A comparison of normality testing methods by empirical power and distribution of *P*-values. Commun Stat. 2021. 10.1080/03610918.2021.1963450.

[35] Torabi H, Montazeri NH, Grané A. A test for normality based on the empirical distribution function. SORT-Stat Oper Res Trans. 2016;40(1):55–88.

[36] Urzúa CM. On the correct use of omnibus tests for normality. Econ Lett. 1996;53(3):247–51.

[37] Yap BW, Sim CH. Comparisons of various types of normality tests. J Stat Comput Simul. 2011;81(12):2141–55.

[38] Yazici B, Yolacan S. A comparison of various tests of normality. J Stat Comput Simul. 2006;77(2):175–83.

